# Towards field-resolved visible microscopy of 2D materials

**DOI:** 10.1515/nanoph-2024-0707

**Published:** 2025-03-07

**Authors:** Daewon Kim, Mikhail Mamaikin, Ferenc Krausz, Nicholas Karpowicz

**Affiliations:** 28281 Max Planck Institute of Quantum Optics, Garching 85748, Germany; Ludwig-Maximilians-Universität München, Garching 85748, Germany

**Keywords:** electric-field sampling, field-resolved imaging, 2D materials

## Abstract

The investigation of interaction of light with various materials on the sub-cycle time scale requires field sampling techniques with incredibly high temporal resolution. Electro-optic sampling (EOS) provides the sub-wavelength resolution both in time and space giving the opportunity for ultrafast microscopy to observe response of electrons and quasiparticles in real time. For the frequencies approaching the petahertz scale, the oscillations of light are incredibly hard to resolve. In particular, EOS has not been demonstrated for wavelengths below 700 nm. In this perspective, we discuss the potential extension of EOS to cover complete visible spectrum and the impact that it can give to the nanophotonics and material science. Specifically, we describe how the ultrafast dynamics of quasiparticles in some 2D materials can be tracked using the space-resolved EOS.

## 1 Introduction

The direct access to the electric field of light has opened new ways for attosecond science to track motion of electrons and quasiparticles in various materials [1], [2], [3], [4], [5], [6], [7], [8]. The detailed view on the ultrafast phenomena such as light–matter energy transfer dynamics [9], with the sub-cycle temporal resolution greatly deepens our understanding of the fundamental physical processes. An ultrashort pulse after interacting with medium carries rich information about the matter response that can be retrieved by recording the output electric field.

For a long time, the reconstruction of complete electric field structure of a light pulse including its carrier envelope phase (CEP) has been limited to the wavelengths in the terahertz range. Attosecond streaking has extended it to wavelengths in the near-infrared and visible spectral regions through the generation of high harmonics [10]. Recent advances in the field sampling [11], [12], [13], [14], [15], [16], [17] including electro-optic sampling (EOS) [18], [19] offer easier access to the electric field of light for the frequencies on the near-petahertz scale.

Apart from being highly sensitive [20], [21], EOS has another distinguishing feature. It can provide not only time-varying field oscillations averaged over the spatial dimension, but also complete spatio-temporal information of laser pulses with the full waveform at each point in space. This spatially resolved EOS, also called EOS imaging, is popular in the terahertz community [22], [23] and has been recently shown to operate in the near-infrared spectral range [24] and then in the minor part of the visible region [25].

This work briefly discusses prerequisites for the extension of EOS to cover complete visible spectral range, and the implications for new experiments in light–matter interaction. Our concept relies on recent advancements in the generation of solitons and broadband ultraviolet (UV) pulses in the hollow-core fibers (HCFs). This in turn gives enormous opportunities for electric-field-resolved studies of various samples that exhibit interesting behavior when exposed to a visible pulse lasting just a few femtoseconds. Here, we discuss two possible applications: studying the excitonic and polaritonic behaviors in the transition-metal dichalcogenide (TMDC) and hybrid TMDC structures.

## 2 Electro-optic sampling

### 2.1 Basic principles

EOS utilizes the changes in the polarization state of a linearly polarized ultrashort probe pulse in a nonlinear crystal under influence of the electric field of light to be measured, referred to as a test pulse. When the test electric field is considered as quasi-static relative to the duration of the probe pulse, like in the case of the terahertz fields, the polarization rotation can be described through the Pockels effect. When detecting the rapidly oscillating electric fields approaching the petahertz scale, the nonlinear interaction inside the crystal must be treated through the sum- or difference frequency generation between the test and probe pulses. Typically, the sum-frequency generation (SFG) signal is phase-matched, while the newly emitted pulse spectrally overlaps with the probe, with orthogonal polarization. Their projection onto common axes leads to the interference on a detector, e.g. balanced photodiodes. Reading out the intensity as a function of the time delay between the probe and test pulses, a signal proportional to the test electric field can be recorded.

**Figure 1:**
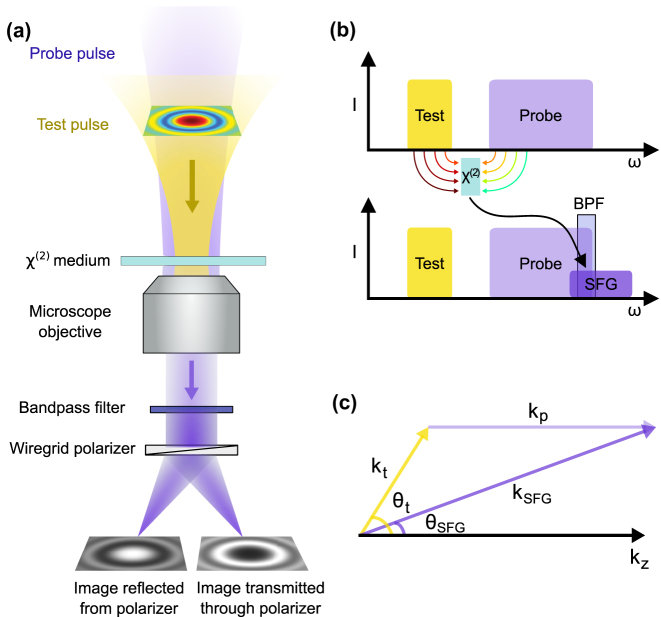
Simplified schematic of EOS imaging. (a) The test and probe pulses are incident on a nonlinear medium, where sum frequency generation (SFG) takes place. The SFG and probe pulses are spectrally filtered to isolate the overlap region and projected by a wire-grid polarizer. The reflected and transmitted beams produce two cross polarized images on a camera. (b) The frequency domain representation of EOS. (c) The momentum conservation allows for a significant reduction of the numerical aperture required to image the test electric field.

To obtain the far-field spatial information of the test electric field, a simple imaging system is set up to image the nonlinear interaction in the crystal as shown in Figure 1. At the same time, the photodiodes that integrate over the active area, must be replaced with a two-dimensional sensor, e.g. a camera. Two images with mutually orthogonal polarizations, obtained by the projection of SFG and the probe using a thin wire-grid polarizer, are subtracted and normalized to get the test field.

The near-field domain can also be accessed by depositing a sample of interest directly on the EOS crystal [24], [26]. This way, the near-field and evanescent radiation is upconverted and carried to the camera provided that the EOS crystal is sufficiently thin. As a result, the spatial resolution is defined by the diffraction limit of the upconverted components (SFG) rather than the wavelength of the test field. It is worth noting that the sub-diffraction-limit resolution of the imaging system is achieved in the wide-field geometry without raster scanning enabling microscopic observations in real time.

### 2.2 Extension to optical band

The temporal convolution involved in detection allows only field oscillations with a half-period less than the duration of the probe pulse to be detected. However, careful spectral filtering of light after the EOS crystal can overcome this limit [18]. This can be explained in detail in the frequency domain. In EOS, the spectral component of SFG (*ω*_*test*_ + *ω*_*probe*_) must interfere with the preexisting spectral component of the probe (*ω*_*probe*_). Thus, isolating only the probe frequencies that overlap with the SFG improves the sensitivity of the detection [27] and enables dramatic extension of the cut-off frequency of EOS [18]. One can conclude that the highest detectable frequency equals to the greatest difference between two frequencies within the probe pulse, Δ*ω*_*probe*_. This means that the bandwidth of the probe and its compression are the main factors that define the spectral limits of EOS.

The EOS and its imaging configuration have been demonstrated across the entire infrared spectral range. Currently, the shortest wavelength detected with EOS is 700 nm, which already lies in the optical band [19], [25]. This result has been achieved using the generation of nearly-single-cycle probe pulses with spectrum spanning from 300 nm to 600 nm. Such visible-UV spectral components can be produced, for example, via self-phase modulation (SPM) and self-steepening effects in gas-filled HCFs. The resulting positive group-delay dispersion of the output pulse is typically compensated using chirped-mirror compressors. Although even shorter wavelengths for the probe can be generated in the same way, the compression of frequencies above 1 PHz over nearly an octave of the bandwidth using chirped mirrors is impossible due to close electronic resonances of the majority of dielectrics.

An alternative and straightforward approach for producing broadband single-cycle UV laser pulses has been recently proposed. It turned out that a conventional hollow-core capillary pumped with high-energy laser pulses can be utilized for both spectral broadening and their direct compression. This intriguing effect is based on the soliton self-compression in the gas-filled HCFs. Typically, the total dispersion of the fundamental mode in a gas-filled HCF is positive, but under some conditions becomes negative for broad bandwidth. This was experimentally observed in hollow-core photonic-crystal fibers more than a decade ago [28], [29] and lately has also been demonstrated in a standard large-core HCF [30]. The nonlinear pulse dynamics inside the capillary drives the generation of high-order solitons and the emission of the resonant dispersive wave (RDW). This enables the creation of self-compressed sub-cycle pulses spanning over multi-octave bandwidth. The RDW represents high-frequency radiation emitted close to the soliton breaking up point, with a relatively high energy conversion from the soliton due to the phase-matching conditions. The wavelength of the RDW can be tuned from the vacuum UV to the visible by changing the dispersion relation using different parameters of the fiber system. Therefore, the soliton and RDW dynamics in the HCFs is highly promising for generation of a probe pulse for EOS in the deep-UV to UV spectral range. This in turn would allow EOS to extend its detection limits to cover complete visible spectrum. The fundamental temporal and spatial resolutions of EOS in this case are expected to be nearly 0.6 fs and 200 nm, respectively.

## 3 Applications

Exploring light–matter interactions can reveal exotic physical behaviours in materials. After the emergence of two dimensional (2D) materials, their different variations such as graphene, hexagonal boron nitride and TMDC are proved to be useful in optoelectronics, nanophotonics, valleytronics, quantum material and topological photonics [31], [32], [33], [34], due to their novel properties and enormous responses in terms of the electronic properties, superconductivity and nonlinearity compared to conventional bulk materials [35], [36], [37]. In particular, TMDCs with chemical formula MX_2_ (M = Mo, W; X = S, Se) are interesting when interacting with pulses in the visible spectrum since their direct bandgap in the monolayer is close to photon energies in the range of visible to near-infrared spectrum [38]. Building on these properties, we will discuss excitonic behaviors in the TMDC van der Waals (vdW) heterostructure and polaritonic effects with each different quasiparticle interaction. EOS imaging can strongly facilitate studying such effects providing subwavelength resolution in space and time.

### 3.1 Excitons in TMDC vdW heterostructure

The energy transfer from photons to electrons in semiconductors can lead to the excitation of an electron from the valence band to the conduction band creating an electron-hole pair. The electron and the hole, attracted to each other by the Coulomb force, can result in a quasiparticle, an exciton, which demonstrates bosonic quantum phenomena. Compared to most bulk materials, the excitons in semiconducting 2D materials emerge even at room temperature and have strongly enhanced exciton binding energy screening of the Coulomb interactions outside the material [39]. Moreover, tailoring this binding energy and the spatial distance between electron and hole, which dominates the optical properties of the material is feasible by engineering 2D materials in various ways [40].

**Figure 2:**
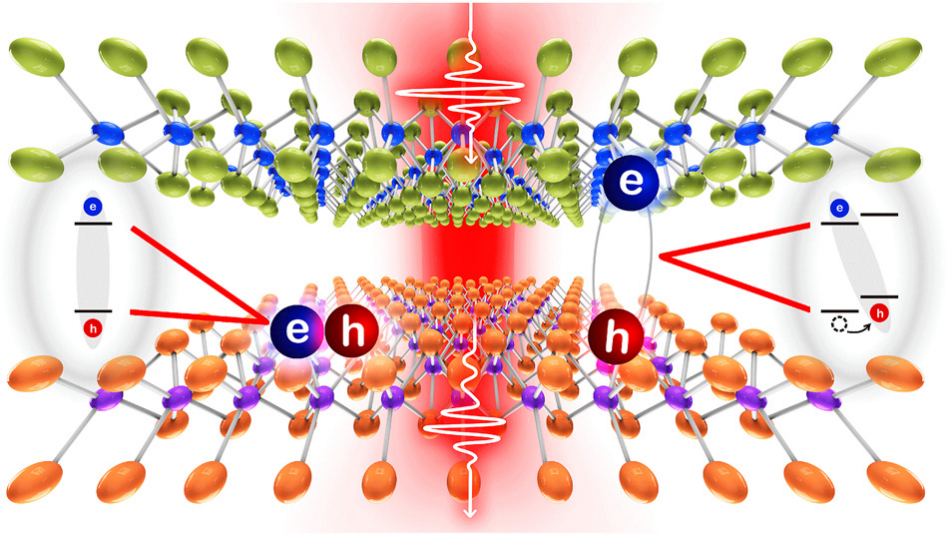
Schematic illustration of observing intra- (left) and interlayer (right) excitons in vdW heterostructures by illuminating the optical pulse. The type-II band alignment in vdW heterostructures enables excitons to spatially separate, with electrons and holes residing in different layers.

One of the marvellous advantages of 2D materials is the possibility to construct vdW heterostructures, which can be stacked respectively without strictly considering the lattice constant unlike bulk materials [41]. Moreover, these layers can be stacked at an angle and paving the way for twistronics to observe moiré pattern, which has exotic properties in physics by introducing different types of patterns what general monolayer materials cannot be formed itself [42]. In addition, TMDC heterostructures can create different types of excitonic behaviors, which come from intralayer and interlayer excitons. Intralayer excitons, which are created in the conduction and valence band of the same material can be formed in one of the layers of the TMDC heterostructure, like the conventional exciton is created in bulk materials. However, since the most TMDC heterostructures have a type-II band alignment, where the conduction band minimum and valence band maximum are located in the different monolayers, the carriers can move to another band and create interlayer excitons (Figure 2) [43].

The exploration of optical and optoelectronic properties of these materials helps to better understand exciton physics. Observation of ultrafast exciton formation in a monolayer of WSe_2_ and chasing ultrafast 1s-2p transition between exciton phases in vdW heterostructures are reported through the near-infrared pump – mid-infrared probe measurement [44], [45]. However, since the light–matter coupling of the TMDC is mostly based on the exciton resonances in the visible to near-infrared region, extending the wavelength of the light source into the visible range can lead to observing high-order energy states transition, i.e. excitonic Rydberg series, with high temporal resolution [39]. Furthermore, the short wavelengths can reveal the information of the trap state dynamics. As a result, the quality of the sample can be determined by observing the defect density [46]. Moreover, interlayer excitons in the twisted vdW heterostructures are affected by the dipole–dipole repulsion and moiré periodic potential. These intriguing properties can be observed in the visible wavelength [47]. In addition to the sub-wavelength temporal resolution, the spatial approach of the EOS can play an important role in imaging a localized exciton in the 2D materials. Imaging the strain, dephasing, and inhomogeneity enables tracking the dephasing time, coupling strength, and interlayer exciton lifetime, which are feasible even in a large area sample [48]. Not only the temporal resolution, but also the spatial analysis is crucial to understand the ultrafast excitonic behavior making the advanced materials to be applied in the realm of the quantum information.

### 3.2 Polariton propagation in TMDC hybrid structures

Light–matter coupling in these materials can also result in the formation of polaritons. These are the quasi-particles arising from mixing photon modes and excitation of a material resonance [49]. The important feature of the polariton is the strong confinement of electromagnetic fields with large momentum in a small scale [50]. In particular, since the TMDCs have robust excitonic behaviors, these exciton resonances can generate strong and stable polaritons by coupling with photon, which is called exciton–polariton [51]. In addition, the exciton–polariton can interact with plasmon and as a result, the plasmon–exciton–polariton can emerge [52]. Here, we discuss one of the measurement methods of exciton–polariton transportation in the MoSe_2_ waveguide [53], spatio-temporal tracing of Rabi-splitting dynamics in the plasmon–exciton–polariton and metasurface-assisted plasmon–exciton–polariton [54], [55].

#### 3.2.1 Exciton–polariton transport in MoSe_2_ waveguide

**Figure 3:**
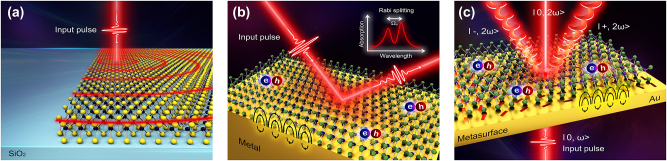
Schematic illustrations of (a) exciton–polariton fringes from the interference between the surface reflection and the scattering from the MoSe_2_ waveguide based on [53]. (b) Exciton–plasmon–polaritons (plexcitons) at a TMDC and Au interface based on [58]. Rabi oscillations and Rabi splitting resulting from the strong coupling between plasmons and excitons can be observed. (c) Metasurface-enhanced exciton–plasmon–polaritons in the Au Pancharatnam–Berry (PB) phase metasurfaces based on [54]. The metasurface facilitates the generation of plexcitons, enabling the encoding and retrieval of valley information through the polarization states.

A stable exciton–polariton (EP) in the microcavity system is operated at cryogenic temperatures, since the lifetime of the polariton starts to decrease at high temperatures [56]. To remove these constraints, some methods for observing the EP at room temperature have been reported [53], [57]. One approach is exploiting waveguide EPs along the 2D material by detecting fringes using a scattering-type near-field scanning optical microscopy from the surface of the sample. The fringes arise from the interference between the surface reflection of the TMDC and the scattering of its waveguide modes (Figure 3(a)). From these fringes, the propagation length of EP is measured. Once mapping the field of these fringes using spatio-temporal EOS imaging is feasible, one can detect the fringes over a large area at room temperature. Since the Fourier transform of the spatial domain yields the momentum (*k*) domain, allowing access to interactions between photons of the waveguide and excitons of the TMDC, and even dispersion relation of the waveguide EP [53]. Moreover, the polariton wavelength lies within the visible spectrum, therefore the significance of the visible-range electric fields is emphasized.

#### 3.2.2 Plasmon–exciton–polariton

Plasmons are the quantization of plasma oscillations, usually in metals due to their large density of free electrons. When light impinges on the metallic nanostructures and the energy of the incident light and the plasmon are matched at a specific incident angle or wavelength, the localized surface plasmon resonance (LSPR) occurs, enabling the electromagnetic field enhancement [59]. Since the resonance frequency of the LSPR depends on the size of the particle, subwavelength nanoparticles induce strong non-propagating plasmon resonance. In addition, at the metal–dielectric interfaces, plasmons can induce propagation of electromagnetic waves, which is called surface plasmon polariton (SPP). Such exotic properties of plasmons are widely utilized in plasmonics and nanophotonics.

One remarkable feature of the surface plasmons is their possibility of coupling to excitons (Figure 3(b)). Since plasmonic modes can effectively mediate the energy transfer range of an exciton [60], the transportable length of the excitons is much longer than its usual diffusion length. When the coupling between the exciton and plasmon is coherent and their energy exchange occurs earlier than the respective decay times, the plasmon–exciton–polariton (plexciton) is formed and the energy can propagate more than conventional coherent length of plasmons [61].

Strong coupling between plasmons and excitons splits two different branches in optical spectra, which are upper – and lower polaritonic branches, separated by Rabi splitting. It depends on the coupling strength between plasmons and excitons. To calculate Rabi splitting energy (*ℏ*Ω_*R*_), a coupled harmonic oscillator model can be used [62]

$$E_{\mathrm{plexciton}}^{U,L}\left(k\right)=\frac{E_{pl}\left(k\right)+E_{ex}}{2}\pm \frac{1}{2}\sqrt{{\left(\hbar \Omega_{R}\right)}^{2}+{\left(E_{pl}\left(k\right)-E_{ex}\right)}^{2}},$$

where $E_{\mathrm{plexciton}}^{U,L}\left(k\right)$ energies of upper (*U*) – and lower (*L*) polaritonic states, $E_{pl}\left(k\right)$ the energy of noninteracting plasmons and *E*_*ex*_ the energy of excitons. From this relation, we can also estimate the period of the Rabi oscillation, *T* = 2*π*/Ω_*R*_, and the Rabi splitting energy in the plexciton has milli-eV scale. It indicates that the ultrafast energy transfer between plasmons and excitons can be on the femtosecond scale and reported to be around 5–10 fs [63]. Since the optimal resonances of exciton formation in TMDC and the surface plasmon in gold are in the range of the visible regime, the Rabi splitting can also be observed in the visible region. The spatio-temporal sampling of the visible electric fields would unprecedently facilitate revealing plexcitonic behaviour by temporally chasing the dynamics of Rabi splitting and spatially observing the hotspot of the localized field distribution in the plexciton systems.

#### 3.2.3 Metasurface-assisted plasmon–exciton–polariton

In the band structure of the semiconducting TMDC, the vertices of the conduction band minimum and valence band maximum in the Brillouin zone (BZ) are called valleys. The valley degree of freedom of an electron corresponds to the specific valley (e.g., +*K* or −*K*) in the BZ where the electron resides. So the electronic properties at the edge of the first BZ of the TMDC are affected by +*K* and −*K* valley points and such properties can be interpreted like the up and down spin or binary number 0 and 1 [64], so it can be used for logic or information storage. Excitons can also be involved in the valleytronics because the binding between electron and hole occur in different valleys. Since monolayer TMDCs break inversion symmetry and exhibit strong spin–orbit interaction, they support spin-valley locking at the +*K* and −*K* points. As a result, it supports two different types of excitons, with different opposite Berry curvatures. However, the short lifetimes of excited valley states and coherence of valleys pose a challenge at room temperature [56].

To remove these constraints, the use of optical metasurfaces has been proposed [65]. Based on the concept of Au-WS_2_ metasurface shown in [55], plasmonic metasurfaces support a photonic spin-Hall effect, which can spatially separate different spin components distinguishing the photon spins and applying the geometric phase to the light (Figure 3(c)) [66]. Moreover, when the surface plasmon polariton from the metasurface interacts with excitons of the TMDC in the evanescent field, the spin-dependent emission from the valley excitons can transfer information of valleys to where excitons are mostly formed [67]. By making nanohole arrays of the metasurface control the polarization of the light, we can induce the Pancharatnam–Berry phase gradient, allowing to control different directions of the different valley information by polarization states [55], [68]. In addition, field enhancement of the plasmonic metasurface can enhance the second harmonic generation (SHG), and these features can replace nonlinear crystals substrate for EOS with the hybrid metasurface sample itself. Finally, with these properties, the nonlinear valley-exciton-locked emissions can be steered in free space at room temperature.

In summary, the plasmonic metasurface can interact with the exciton, enabling the possibility of plexciton formation with enhanced nonlinearity. Furthermore, it can give the location information of where the excitons mainly formed and induce the emission of valley excitons with different polarization states and its direction. The electric field of a visible pulse is advantageous, to match the natural frequency of these effects. Moreover, enhancement of the spatial resolution such as using non-invasive tip-enhanced methods [69], [70] will pave the way for observing the localized valley excitons in the metasurface-assisted TMDC structures.

## 4 Conclusions

In conclusion, we have explored the implications of visible-light EOS in emerging frontiers of nanophysics. In particular, imaging capability of EOS would enable space-resolved detection of quasiparticles such as excitons, polaritons, plasmons and their interactions. Here, we discussed a few examples of conceptual measurements of such quasiparticles in the 2D materials that recently have attracted great attention. We believe that field-resolved microscopy of light–matter interactions will greatly facilitate research in nanophotonics and material science.
